# Biorisk Management in High-Containment Laboratories: Assessment of Certification Systems and Need for Global Standardization

**DOI:** 10.3390/pathogens15090951

**Published:** 2026-09-07

**Authors:** Anna Rosa Garbuglia, Verdiana Zulian, Silvia Pauciullo, Daniele Lapa

**Affiliations:** Laboratory of Virology and Laboratories of Biosafety, National Institute for Infectious Diseases “Lazzaro Spallanzani”—IRCCS, 00149 Rome, Italy; verdiana.zulian@inmi.it (V.Z.); silvia.pauciullo@inmi.it (S.P.)

**Keywords:** biosafety, biosecurity, biorisk, high-containment laboratories, standardization, certification, accreditation, BSL-3, BSL-4

## Abstract

Laboratory-acquired infections (LAIs) and accidental pathogen escape from laboratory settings (APELS) are still a significant concern, even with biosafety guidelines and manuals in place. The actual number of these events is probably underestimated because there are no mandatory reporting systems or standard surveillance frameworks. In this review, a clear overview is given about the evolution of biosafety, the worldwide distribution and governance of high-containment laboratories (BSL-3 and BSL-4), and the key factors that lead to laboratory incidents. The focus is specifically on human error, inadequate training, and differences in standard operating procedures as primary causes of biosafety failures. Furthermore, current regulatory frameworks are described, pointing out the lack of consistency in laboratory practices, infrastructure needs, and national policies, especially the variances in biosafety level recommendations for high-risk pathogens. Certification systems as potential tools to improve standardization and consistency in operations are discussed. Among these, ISO 35001 is a detailed standard for managing biorisks, including risk assessment, staff training, incident reporting, and processes for ongoing improvement. However, its use is still limited, and certification is not mandatory for laboratory licensing everywhere. Additionally, existing standards do not fully cover structural aspects and national differences in how pathogens are handled. This review also highlights the limitations of current certification systems and proposes recommendations to strengthen and harmonize standardization procedures across high-containment laboratories. Overall, this work highlights the need for greater global consistency in biosafety practices, suggesting the establishment of an independent international auditing body as an important step toward enhancing global health security and laboratory readiness.

## 1. Introduction

**Figure 1:**
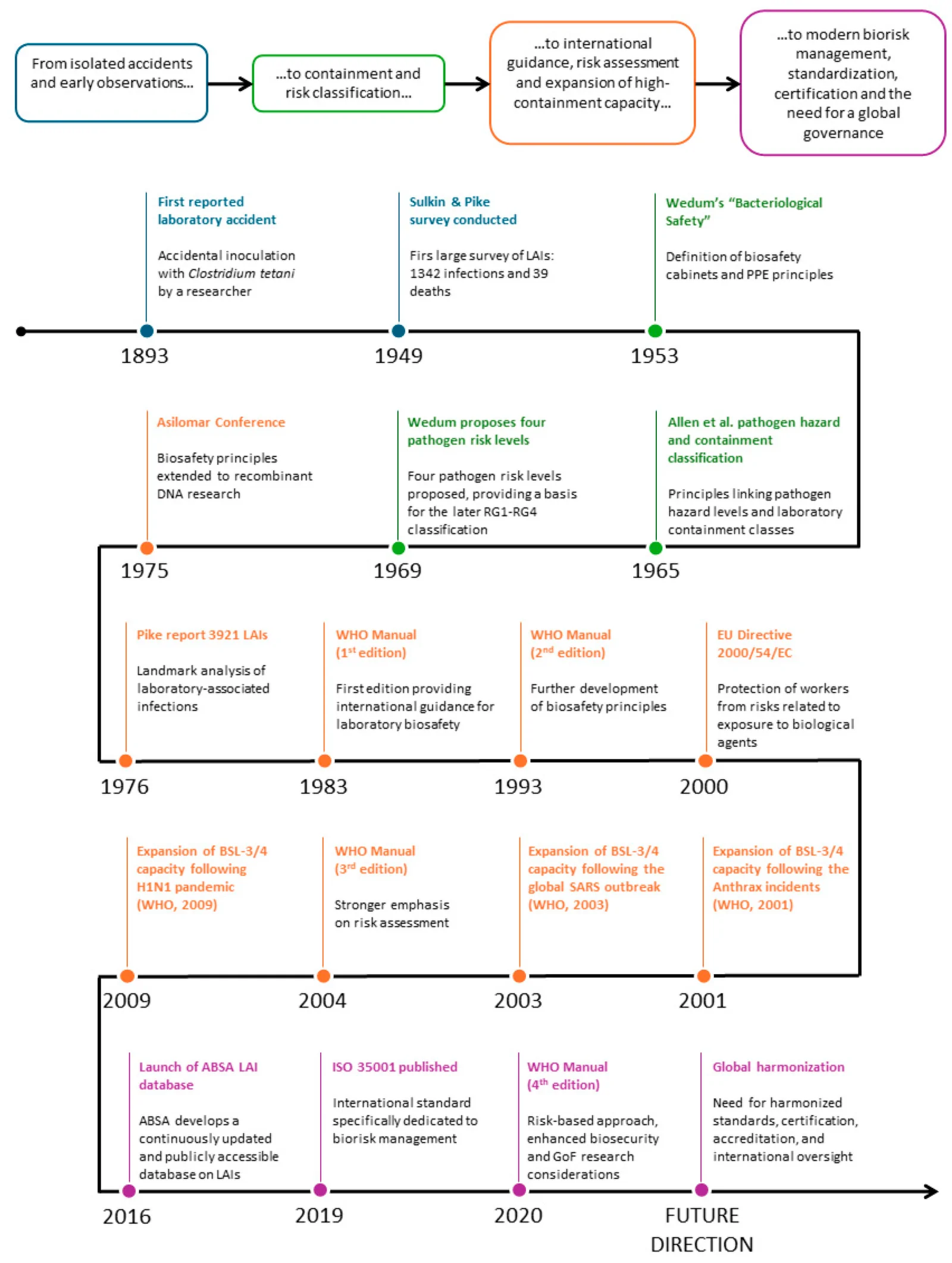
Historical evolution of laboratory biosafety and biorisk management. The timeline summarizes major milestones from the first reported laboratory accident in 1893 to modern risk-based biorisk management and international standardization. The final element indicates the future need for harmonized standards, certification, accreditation, and international oversight. LAI, laboratory-acquired infection; BSL, biosafety level; PPE, personal protective equipment; RG, risk group; GoF, gain-of-function; WHO, World Health Organization; EU, European Union; SARS, Severe Acute Respiratory Syndrome; ABSA, American Biological Safety Association; ISO, International Organization for Standardization. WHO, 2001: https://www.who.int/emergencies/disease-outbreak-news/item/2001_10_18-en (accessed on 22 August 2026); WHO, 2003: https://www.who.int/emergencies/disease-outbreak-news/item/2003_04_11-en (accessed on 22 August 2026); WHO, 2009: https://www.who.int/emergencies/situations/influenza-a-%28h1n1%29-outbreak (accessed on 22 August 2026).

Laboratory accidents affecting personnel, the environment, and the wider community have been investigated for many years. However, due to the lack of mandatory reporting systems and the absence of penal or administrative sanctions in case of non-notification, the real number of such events is likely underestimated. In this paper, after a brief historical overview of the most significant laboratory accidents reported in the literature and of surveys on these accidents that highlighted the magnitude of the problem, we describe the main regulatory acts that contributed to defining the field of biosafety and biosecurity (Figure 1). We underline that a real standardization is still lacking, both in terms of management practices and minimum infrastructure requirements [1]. Among the available tools to verify proper laboratory performance and correct operational procedures, certification systems play a key role. In particular, ISO 35001 certification, specifically designed for biorisk management, is described in more detail, highlighting its contribution in preventing both operator-related incidents and those linked to structural or organizational inefficiencies.

The first laboratory accident reported in the literature dates back to 1893, when a researcher accidentally inoculated himself with *Clostridium tetani* [2,3]. Other incidents, mainly associated with bacterial pathogens such as *Coxiella burnetii* (Q fever), *Salmonella typhimurium*, and *Brucella melitensis*, were described during the first half of the 20th century [4,5].

The first large survey on laboratory accidents was conducted in 1949, when Sulkin and Pike distributed questionnaires to approximately 5000 laboratories, including hospital, university, veterinary, and industrial laboratories producing biological products. In that survey, 1342 laboratory-acquired infections (LAIs) [6] were identified, caused by 69 different pathogens, among bacteria, viruses, fungi, and parasites with 39 reported deaths [7]. These findings already pointed out the need for clearer guidelines in pathogen handling, both in clinical practice and research activities.

In 1953, Wedum published the milestone paper *Bacteriological Safety*, where different biosafety cabinet levels (including Class I and Class III) were defined, together with other containment equipment (animal housing equipment, sealing centrifuge sleeves, shakers) [8]. Wedum also emphasized the importance of personal protective equipment (PPE), such as gloves and masks, especially when handling airborne pathogens. These aspects were later discussed in further studies focusing on airborne transmission risks [9,10].

In 1965, Allen and colleagues introduced a classification system defining pathogen hazard levels (from A, non-pathogenic to man or animals, to H, extremely pathogenic to man), as well as the containment level of laboratories from class 1 to 3 (minimal to maximal containment) [6]. They also established that biosafety levels for studies involving Class E–G pathogens must be approved by the biohazard committee and highlighted the responsibility of the principal investigator, underlining the importance of safety management, personnel training, accident procedures, and regular inspections [6]. In 1969, Wedum published the “Assessment of risk of human infection in the Microbiology Laboratory” and proposed four levels of risk attributable to a given pathogen, from Level 1 (low risk) to Level 4 (highest risk), a classification later confirmed by the U.S. Public Health Service [11,12]. Subsequently, this pathogen classification has been accepted by the National Institutes of Health (NIH), the Centers for Disease Control and Prevention (CDC), and the World Health Organization (WHO), which define the risk groups as follows:

1. Risk Group 1 (RG1): Agents that are not associated with disease in healthy adult humans. This group includes a list of animal viral etiologic agents in common use. These agents represent no or little risk to an individual and no or little risk to the community.
2. Risk Group 2 (RG2): Agents that are associated with human disease which is rarely serious and for which preventive or therapeutic interventions are often available. These agents represent a moderate risk to an individual but a low risk to the community.
3. Risk Group 3 (RG3): Agents that are associated with serious or lethal human disease for which preventive or therapeutic interventions may be available. These agents represent a high risk to an individual but a low risk to the community.
4. Risk Group 4 (RG4): Agents that are likely to cause serious or lethal human disease for which preventive or therapeutic interventions are not usually available. These agents represent a high risk to the individual and a high risk to the community [13,14,15].

Risk group classification is based on several pathogen-related characteristics, including pathogenicity, disease severity, transmissibility, and the availability of effective preventive or therapeutic measures. More detailed information on the classification of specific pathogens, particularly those assigned to RG3 and RG4, is provided in the relevant international guidelines [16,17,18]. Importantly, assignment to a risk group does not automatically determine the Biosafety Level required for a specific laboratory activity, which should be established through a procedure-specific risk assessment and consideration of the characteristics of the biological agent and the type of manipulation performed.

In 1975, during the Asilomar Conference organized by Paul Berg, for the first time safety issues related to recombinant DNA experiments were discussed, stressing the need for standardized biosafety procedures for the handling of both genetically modified and non-genetically modified microorganisms [19]. At that time, the concept of biosafety—intended as safety in biological containment—was clearly framed as the need to protect both operators and the environment from accidental exposure [20].

In 1976, Pike updated the data on LAIs, reporting 3921 cases—2370 due to bacteria and 1049 to viruses—with a mortality rate of approximately 4.4%, with more than 50% occurring in research laboratories [21].

In 1983, WHO published the first edition of the Laboratory Biosafety Manual, providing detailed indications on BSL-3 and BSL-4 laboratory requirements, pathogen transport and waste disposal [22]. For example, BSL-3 laboratories must be physically separated from laboratories carrying out different activities and should be equipped with double-door entry systems, including airlocks and buffer zones. These facilities have, as engineering controls, negative pressure conditions, with HEPA air filtration systems capable of preventing pathogen escape [22].

The WHO manual represented a fundamental attempt toward standardization. The second (1993) and third (2004) editions further refined the concept of biosafety, defined as “The containment principles, techniques, and practices implemented to prevent accidental release” [23,24]. Furthermore, the third edition placed particular emphasis on risk assessment procedures for the protection of personnel and community [24]. Finally, the fourth edition (2020) introduced additional biosecurity recommendations aimed at ensuring safe and secure handling of materials with high biological risk and included a section dedicated to Gain of Function (GoF) research [16]. GoF generally refers to changes resulting in the acquisition of new biological phenotypes or the enhancement of existing ones [25].

Moreover, some countries, such as China, have adopted specific biosafety regulations. For example, in 2004, the State Council issued the Regulations on Biosafety Management of Pathogenic Microorganism Laboratories, defining supervisory criteria, procedures for pathogen handling, and legal responsibilities in cases of non-compliance [26]. These regulations were further updated between 2016 and 2018 with more analytical indications, especially regarding personnel safety.

In the meantime, Harding and Byers produced a comprehensive analysis based on literature data, including descriptions of LAIs reported in case reports and other scientific papers between 1979 and 2004, later updating the dataset up to 2015 in the United States. The authors observed that many LAIs were due to accidental exposures and frequently occurred in the field of veterinary research, generally with low consequences for the operators [27,28]. During the period 1979–2004, data showed that bacterial LAIs were more commonly observed in clinical laboratories (n = 471) compared to research laboratories (n = 84). In contrast, incidents related to viral manipulation showed an opposite trend, with 418 cases occurring in research laboratories and 181 in clinical laboratories [29]. In the following years, LAIs associated with viral agents remained predominant in research laboratories, representing about 67%, whereas only around 10% of LAIs in these settings were linked to bacterial agents [28]. Since 2016, the ABSA International (American Biological Safety Association) has developed a continuously updated and publicly accessible database on LAIs [30].

## 2. Laboratories BSL-3 and BSL-4

### 2.1. Overview of the Distribution and Management of BLS-3 and BSL-4 Laboratories

The number of high-containment biological laboratories (HCBLs), especially BioSafety Level (BSL)-3 and BSL-4 facilities, increased after the events of 18 September 2001, when an anthrax-related incident caused four deaths. Later pandemics, such as SARS-CoV in 2003 and H1N1 in 2009, further reinforced the need for research on high-risk pathogens (Groups 3 and 4) for developing diagnostics, vaccines and antiviral strategies [31]. The main operational, containment, and structural differences between BSL-3 and BSL-4 laboratories are summarized in Table 1.

Although the expansion of these facilities is essential for public health preparedness, inadequate management can introduce biosafety concerns to reduce exposure and involuntary release of high-level pathogens. Many BSL-3 and BSL-4 laboratories have been built without a consistent global oversight. In fact, Many BSL-3 and BSL-4 facilities operate within heterogeneous national regulatory systems, in the absence of a consistent and binding international oversight framework, as highlighted by Muluneh et al. and Lentzos and Koblentz [33,38]. Furthermore, to date there is no comprehensive international registry of existing BSL-3 and -4 laboratories. Muluneh reported the presence of 3515 BSL-3 laboratories across 149 countries, with nearly half (47.1%) located in the United States. The 110 identified BSL-4 facilities are distributed across 34 middle- and high-income countries, with 46% situated in the WHO European Region [33]. Notably, 91.6% of countries hosting at least one BSL-3 laboratory lack guidelines for dual-use research of concern [33].

Considerable discrepancies exist between sources about BSL-4 laboratories. For example, following the completion of the project promoted by King’s College London (Global Biolabs), a report published in 2023 indicated the existence of 69 BSL-4 laboratories, either operational or under construction, across 27 countries [39]. In contrast, the World Health Organization reported only 43 BSL-4 laboratories worldwide [40].

In the United States, the Federation of American Scientists (FAS) reported that in 2008 there were 12 BSL-4 laboratories, two of which had an unconfirmed status and three under construction [41,42]. Similarly, Gronvall et al. reported in 2007 the presence of 11 BSL-4 laboratories, including four under construction expected to be completed in 2008 [43]. However, in 2008, the Government Accountability Office (GAO) reported only five BSL-4 laboratories, which corresponds to approximately one-third of the number reported by other sources [44]. These discrepancies are of concern, considering that the selected agents handled in BSL-4 laboratories should be regularly reported to governmental authorities.

Among the 34 African countries reporting at least one BSL-3 laboratory, 32 lack biosafety-related regulations, legislation, or mandatory training requirements [26]. In some of these countries, BSL-3 laboratories have been established with the support of international partners providing technical expertise [45]. For example, in 2015, China supported the government of Sierra Leone in building its first fixed BSL-3 laboratory (as mobile BSL-3 facilities were already in use) to address the Ebola emergency [26,46].

In 2010, a collaboration between the U.S. Defense Threat Reduction Agency and the government of Kazakhstan was initiated, including the construction of a biosafety laboratory dedicated to the research and monitoring of highly pathogenic viruses (Risk Groups 3 and 4) [47]. Similarly, Kyrgyzstan established a BSL-4 laboratory with financial and technical support from the Canadian government [26].

Although these initiatives represent important efforts to strengthen the response capacity to public health emergencies, limited financial resources and insufficient oversight may result in facilities that do not fully meet minimum biosafety standards (e.g., advanced engineering controls, specialized facility design, negative air pressure systems, and rigorous operational protocols to completely isolate high-risk pathogens and prevent any accidental exposure or environmental release), thereby increasing the likelihood of incidents [48].

The Global Biolabs report, which evaluated biosafety and biosecurity levels using parameters such as legal frameworks, risk assessment, incident response planning, risk management, and training, showed considerable variability among countries [39]. Biosafety scores reached values as high as 18/20 in countries such as Australia and Canada but were as low as 1/18 in Saudi Arabia. Similarly, biosecurity scores were 18/18 in the United States and France but only 1/18 in Gabon [39].

With regard to Asian countries, in 2008 only two out of eleven countries hosting BSL-3 laboratories had general biosafety regulations supported by written laboratory policies and standards [49]. Although biosafety guidelines may exist, the absence of effective oversight mechanisms makes it difficult to assess the level of compliance across individual BSL-3 and BSL-4 laboratories.

### 2.2. Laboratory Incidents in High-Containment Laboratories

Despite the development of biosafety guidelines, laboratory incidents continue to occur in diagnostic, academic, and industrial laboratories. In this context, the term APELS (Accidental Pathogen Escape from Laboratory Setting) has been introduced alongside LAIs. While LAIs refer to infections acquired during laboratory activities, APELS include both internal consequences (affecting laboratory personnel) and external consequences (impacting the environment and the wider community) [29]. Both types of events may result from human errors, lack of adherence to procedures, or failures in laboratory infrastructure where pathogens are handled.

A study covering the period from 2000 to 2021 reported 309 incidents, including both LAIs and APELS and involving 51 pathogens, eight of which were fatal [29]. The majority of these events (69.3%) were associated with procedural errors by laboratory personnel. The LAIs were caused by emerging viruses such as Zika virus, and SARS-CoV, whereas APELS involved pathogens risk group 3 (RG3) in the 75%, RG2 in 18.8%, and RG4 in 6.6% of cases [26].

Several incidents related to human error or non-compliance with procedures have been reported over the last two decades. In 2003, six documented SARS-CoV laboratory escapes from HCBLs occurred in Singapore (one case from BSL-3), Taiwan (one case from BSL-4), and China (four cases from the same BSL-3) [50]. For example, in the Singapore case, contamination of a West Nile virus sample with SARS-CoV-1 was identified, and insufficient training of personnel—including students—poor record-keeping, lack of freezers, HEPA/air filter problems, and malfunctioning equipment were reported [51].

In 2014, several biosafety failures were reported at the CDC: (1) improper inactivation of *Bacillus anthracis* by personnel [52]; (2) cross-contamination of the non-pathogenic influenza H9N2 strain with the highly pathogenic influenza H5N1 strain at the CDC Influenza Division, specifically the Virology, Surveillance and Diagnostics Branch (VSDB) BSL-3 laboratory. The contaminated culture was subsequently sent to an external high-containment laboratory at the U.S. Department of Agriculture Southeast Poultry Research Laboratory (SEPRL), where the contamination was detected only after receipt [53]; (3) improper storage of boxes containing vials, dated from 1946 to 1964, of various biological agents, including dengue, influenza, Q fever, rickettsia, variola virus, and unknown unlabeled samples, in an unsecured FDA storage facility located on the NIH Bethesda campus [54].

In the same year, a case of 4-year-old child infected with poliovirus (Saukett strain, used for anti-polio vaccine production in the USA) was reported in China, although the origin (vaccine contamination or accidental release) remained unclear [55].

Infrastructure failures have also been associated with several laboratory-related incidents. In 2007, an outbreak of foot-and-mouth disease virus (FMDV, serotype O subtype BFS1860 isolated in 1967, a strain non circulating globally but used for vaccine production) in the United Kingdom was linked to leakage from a damaged wastewater pipeline at a high-containment facility, leading to environmental contamination [56,57,58].

In 2014, an accidental release of wild poliovirus type 3 from a vaccine production facility in Belgium occurred, involving contamination of wastewater systems [59].

In 2019, a Brucella outbreak in Lanzhou Veterinary Research Institute (LVRI, China) was associated with the use of expired disinfectants, resulting in aerosol transmission affecting both laboratory personnel and surrounding communities [60,61]. By 30 November 2020, the Health Commission of Lanzhou reported 10,528 cases [60].

Overall, human error still represents a substantial proportion of laboratory incidents. Many LAIs and APELS can be attributed to insufficient training, limited experience, inadequate knowledge of standard operating procedures (SOPs), or a combination of these factors. This issue appears particularly relevant in academic laboratories, where safety training is often less structured and staffed by students vs. professional laboratory trained technicians with the appropriate certifications [62].

Indeed, the incidence rate in academic settings has been estimated at approximately 2.5 events per week in BSL2 [63]. Experimental studies using fluorescent tracers reported error rates ranging from 1 to 26 errors per 1000 pipetting steps, with variability depending on operator experience [64].

A survey has also highlighted significant gaps in safety knowledge, especially among students. Only about 43% of students correctly answered questions regarding laboratory ventilation compared with 69% of laboratory workers [65]. Furthermore, a proportion of young researchers or fellowship (2.1–16.9%) did not consistently follow basic safety practices, and only a limited number (12.7%) were familiar with emergency safety equipment [65,66].

Finally, differences in the design of standard operating procedures and in training programs contribute to variability in safety standards between laboratories. Several international networks have been established to promote harmonization of biosafety practices: (1) Global Partnership’s Biological Security Working Group (BSWG), (2) BSL4 Zoonotic Laboratory Network (BSL4ZNET), (3) European Research Infrastructure on Highly Pathogenic Agents (ERINHA). However, their participation is voluntary and therefore not universally implemented across all BSL-3 and BSL-4 facilities [67,68,69].

## 3. Certification, Standardization, and Accreditation

In BSL-3 and BSL-4 laboratories, the distinction between certification, standardization, and accreditation is particularly important because these processes contribute in different ways to the establishment and maintenance of safe and reliable high-containment environments. Certification is concerned with demonstrating that specific facilities, systems, processes, or components conform to defined requirements [70]. Standardization provides the reference framework by defining, through consensus-based standards, the technical, operational, and management requirements relevant to laboratory design, biorisk management, and biosafety practice [71]. Accreditation provides a further level of assurance by formally recognizing that a laboratory, or a conformity assessment body, possesses the technical competence, impartiality, and organizational capacity to perform defined activities reliably and consistently [70,72]. Although these concepts are closely related, they are not interchangeable [70,71,72]. Rather, they operate as complementary components of a broader assurance framework: certification demonstrates conformity with established requirements; standardization defines those requirements and provides the relevant reference framework; and accreditation formally recognizes the competence and impartiality of the organizations responsible for the relevant conformity assessment activities.

### 3.1. Certification

In the context of BSL-3 and BSL-4 laboratories, certification is the process of verifying that a facility, system, process, or component conforms to defined biosafety, engineering, operational, or regulatory requirements [16,70]. It is closely associated with commissioning, periodic revalidation, and authorization to operate.

In BSL-3 laboratories, this commonly includes the assessment of directional airflow, pressure differentials, ventilation performance, HEPA filtration where required, biological safety cabinets, alarms, access control, decontamination systems, waste-management procedures, emergency plans, SOPs, personnel training, maintenance records, and documentation systems [16,73,74,75,76,77,78]. In BSL-4 laboratories, the assessment is more stringent and may include dedicated or redundant air-handling systems, HEPA-filtered exhaust, physical separation from other building systems, validated personnel and material decontamination, effluent treatment, positive-pressure suit systems where applicable, inventory control, biosecurity measures, emergency power, containment-envelope integrity, and failure-condition verification of Heating, Ventilation and Air Conditioning (HVAC) performance [16,78,79,80].

Although specific requirements vary across jurisdictions, they generally derive from a combination of international biosafety guidance, national occupational health and safety legislation, institutional biosafety policies, competent authority approvals, and technical standards [16,73,74,75,76,77,78,79,80,81,82]. Accordingly, the body or professional responsible for certification depends on the applicable national and institutional framework. Certification may involve qualified commissioning agents, biosafety professionals, engineering specialists, independent inspection bodies, equipment certifiers, institutional biosafety committees, or competent national authorities [16,73,74,75,76,77,78,79,80,81,82]. Importantly, no single internationally harmonized certification scheme currently exists for BSL-3 and BSL-4 laboratories.

Facility certification and containment authorization are therefore implemented through different national or regional frameworks. In the European Union, Directive 2000/54/EC provides the overarching framework for worker protection against biological agents [73]. National examples include Legislative Decree 81/2008 in Italy [74], the Arrêté du 16 juillet 2007 in France [75], and the combination of COSHH, ACDP/HSE guidance, and SAPO licensing in the United Kingdom [76,77]. Outside Europe, examples include the Canadian Biosafety Standard in Canada [78], the Federal Select Agent Program and BSL-4/ABSL-4 verification policy in the United States and Biosafety in Microbiological and Biomedical Laboratories (BMBL) guidance [15,83,84], the OGTR certification framework for PC3/PC4 facilities in Australia [81], and certified facility requirements under the biosafety framework in Singapore [82].

### 3.2. Standardization

The main function of standardization is to promote harmonization, comparability, and reproducibility across laboratory settings. Standardization can be developed and implemented at the international, national, or local level. At the international level, ISO-based standards have become central to laboratory governance because ISO standards are internationally agreed documents developed by experts to describe consistent ways of carrying out products, processes, services, or activities (Table 2) [71].

Other organizations, such as the Clinical and Laboratory Standards Institute (CLSI), have also contributed substantially to the development of laboratory standards and guidance, particularly in the medical field [98].

Before ISO-based standards became widely adopted, quality assurance in medical laboratories was largely shaped by national regulatory frameworks and professional practice. In the United States, federal oversight of clinical laboratory quality predates ISO-based standardization and included the Clinical Laboratories Improvement Act of 1967 (CLIA ’67), and the current regulatory framework derives from the Clinical Laboratory Improvement Amendments of 1988 (CLIA ’88), which established federal quality standards for laboratories testing human specimens for health assessment, diagnosis, prevention, or treatment of disease [99,100,101]. CLIA ’88 was introduced to ensure that laboratory results were accurate, reliable, and timely, regardless of where testing was performed [100,101]. In this respect, the U.S. model represented an early national regulatory approach to laboratory quality standards.

In Europe, national quality systems also emerged before the consolidation of ISO-based systems. In France, the Guide de Bonne Exécution des Analyses de Biologie Médicale (GBEA), introduced in 1994 and revised in 1999, represented an early national regulatory document specifically dedicated to quality in medical laboratories [83,84]. Rather than prescribing a single analytical method, the GBEA promoted an organized working methodology intended to ensure the accuracy and precision of laboratory results across all phases of the testing pathway, from specimen collection to result reporting [83,84].

At the international level, ISO/IEC (International Electrotechnical Commission) Guide 25:1990 had already established general requirements for the competence of calibration and testing laboratories [102]. This was later replaced by ISO/IEC 17025:1999, which marked an important step toward harmonized requirements for laboratory competence [102,103,104]. The current version, ISO/IEC 17025:2017 defines the general requirements for the competence, impartiality, and consistent operation of testing and calibration laboratories [88]. ISO/IEC 17025 also provided an important basis for the development of ISO 15189, which was designed specifically for medical laboratories [88,93,104]. Every ISO are subject to periodic review, generally at least every five years, to determine whether they should be confirmed, revised, or withdrawn [105].

Within this broader framework, different standards serve complementary but distinct purposes. A first group includes general laboratory and conformity-assessment standards, which define requirements for quality management, competence, accreditation, inspection, or certification, but are not specific to BSL-3 or BSL-4 facilities. ISO 9001:2015 provides generic requirements for quality management systems and supports process control, customer focus, risk-based thinking, and continual improvement [85]. A central element is the principle of continual improvement through a cycle of planning, implementation, evaluation, and process improvement, commonly referred to as the Plan–Do–Check–Act (PDCA) cycle. The PDCA cycle therefore provides a link between general quality management and biorisk management. This management philosophy, already embedded in ISO 9001 [85], has subsequently been adopted in other management-system standards, including ISO 45001 and ISO 35001 [89,90].

ISO/IEC 17025:2017 establishes the general requirements for the competence, impartiality, and consistent operation of testing and calibration laboratories, and remains one of the principal reference standards for formal accreditation [88]. ISO 15189:2022 is specifically intended for medical laboratories and addresses both quality management and competence, including activities directly related to patient care [93]. Point-of-care testing (POCT) had previously been addressed through ISO 22870:2016, which was intended to be used together with ISO 15189. This standard has now been withdrawn, and ISO 15189:2022 is identified by ISO as the current standard applicable also to POCT. This change reflects a broader consolidation of point-of-care governance within the medical laboratory quality framework [93,106].

Beyond the standards that directly govern laboratory accreditation, a wider set of ISO and ISO/IEC standards helps sustain the broader framework within which laboratory quality and competence are evaluated. ISO/IEC 17011:2017 defines how accreditation bodies must operate, setting requirements for their competence, impartiality, and consistent functioning when assessing and accrediting conformity assessment bodies such as laboratories, inspection bodies, proficiency testing providers, and reference material producers [72].

Within this broader system, ISO/IEC 17043:2023 plays an important role by establishing requirements for proficiency testing providers. Although laboratories are not themselves accredited to this standard, their participation in proficiency testing and external quality assessment schemes operated by competent or accredited providers is essential for comparing performance across laboratories, monitoring analytical quality over time, and demonstrating that results remain valid and comparable [95]. ISO 17034:2016 also supports laboratory quality by applying to reference material producers and defining the conditions under which reference materials, including certified reference materials, are produced [87]. This is especially relevant in laboratory practice because reliable reference materials underpin calibration, method validation and verification, metrological traceability, internal quality control, and the reliability of measurement results [87]. The framework becomes broader still when conformity assessment extends beyond analytical testing itself. In such cases, ISO/IEC 17020:2026 becomes relevant, as it applies to inspection bodies and sets requirements for the competent, impartial, and consistent inspection of materials, products, installations, plants, processes, procedures, and services [96]. This makes it particularly important whenever independent inspection of facilities, systems, equipment, or compliance-related operations forms part of the overall assurance process [96]. Finally, when the focus shifts from organizations to individuals, ISO/IEC 17024:2026 provides the relevant reference point. This standard applies to bodies certifying persons and is therefore concerned with the formal recognition of individual competence under defined certification schemes, rather than with laboratory competence or institutional accreditation [97]. In laboratory-related settings, it is most relevant where the competence of highly specialized professionals, auditors, biosafety personnel, or technical staff must be independently certified [97]. Together, these standards complement laboratory-focused accreditation standards by regulating the external bodies, materials, assessment schemes, inspection activities, and personnel certification processes that support confidence in laboratory quality and competence.

Environmental and engineering control standards are also important. ISO 14644-1:2015 defines air cleanliness classes based on airborne particle concentration in cleanrooms and associated controlled environments, while ISO 14644-3:2019 provides the corresponding test methods for evaluating cleanroom and clean-zone performance under as-built, at-rest, and operational conditions [86,91]. These aspects are highly relevant where containment, filtration efficiency, and clean-air performance are critical [86,91].

In addition, information protection is becoming increasingly important in the governance of high-containment laboratories. ISO/IEC 27001:2022 specifies requirements for an information security management system and may therefore be relevant for laboratories handling sensitive pathogen inventories, research data, access control systems, or other forms of critical biosafety and biosecurity information [94].

Another group of standards support biorisk management, laboratory safety, occupational health and safety, controlled-environment qualification, and information security in settings where hazardous biological agents are handled. In high-containment laboratories, the general quality framework must therefore be integrated with biosafety- and biosecurity-oriented requirements.

This integration is facilitated by a biorisk management-system approach, which enables institutions to identify, assess, control, monitor, and continually improve the management of biosafety and biosecurity risks inherent to their activities.

In line with this risk-based logic, the WHO Laboratory Biosafety Manual, 4th edition, adopts a risk- and evidence-based approach in which biosafety measures are selected on the basis of hazards, procedures, and local context rather than fixed containment categories alone [16]. Within this framework, ISO 15190:2020 specifies safety requirements for medical laboratories and provides a structured basis for safe laboratory practice [92]. ISO 45001:2018 adds a broader occupational health and safety management perspective, offering a formal system for identifying workplace hazards, implementing risk controls, ensuring worker participation, and supporting continual improvement in occupational safety performance [89].

Finally, ISO 35001:2019 provides the first internationally recognized standard specifically dedicated to biorisk management for laboratories and other related organizations, defining a management-system approach based on risk identification, assessment, control, monitoring, and continual improvement [90].

#### ISO 35001 and Biorisk Management System Certification

ISO 35001 is widely considered the most comprehensive certification system for biorisk management including BSL-3 and BSL-4 laboratories. It addresses several aspects in a detailed way, with particular emphasis on risk assessment [90]. Clause 6.1 highlights how risk assessment supports the identification of both biological and organizational risks. It also focuses on the importance of external factors (e.g., emerging pathogens, new regulations) which may influence the system and therefore need to be considered when updating risk assessment procedures. The use of specific tools, such as SWOT analysis, is recommended to facilitate the timely identification of risks and to define possible mitigation strategies [90]. In addition, verification of the effectiveness of implemented actions is required, in order to ensure that certain risks are adequately controlled—for instance, by confirming that no further non-conformities or incidents occur after corrective measures have been applied.

Within the framework of biorisk management, two main steps are identified: (1) the identification of all biological hazards and/or threats that may lead to an incident, and (2) the evaluation of how these hazards or threats could result in a negative outcome. A risk score is then introduced, generally based on a mathematical approach, often using probabilistic models.

The control plan must be included within the risk assessment. Furthermore, the standard specifies that the biorisk management system should be SMART (Specific, Measurable, Achievable, Relevant, Time-bound), as indicated in Clause 6.2, in order to improve clarity and ensure that procedures are easily understood by all personnel.

Clause 6.4 also requires the implementation of an internal risk register, where all incidents must be recorded, regardless of whether they are caused by human error or infrastructural failures. Each risk should be assigned a score. The standard also provides guidance on risk evaluation methods, for example using the BOWTIE diagram, which can support a more standardized approach to risk assessment.

In high-containment settings such as BSL-3 and BSL-4 laboratories, monitoring and surveillance are particularly relevant. These include routine checks of air quality, surface swabbing to detect possible contamination, and the use of biological indicators to verify the effectiveness of sterilization and decontamination procedures [90].

Clause 7 focuses on the need for adequate resources to ensure proper laboratory management, especially in biosafety facilities. Continuous training of personnel is considered essential. Staff must be regularly informed about procedural updates, reported incidents, and the measures taken to address them. ISO 35001:2019 requires documentation including policies, plans, procedures, protocols, and records.

Documentation related to decontamination processes must be maintained and regularly updated (Clauses 7.5 and 7.8), and decontamination procedures must be validated (Clause 8.1), as part of operational planning and control.

Compliance with local biosafety regulations is mandatory. Any modifications to existing facilities or the construction of new laboratories must be performed according to an approved plan. External providers responsible for services such as autoclave maintenance, waste management, and courier transport must also be monitored (Clause 8.3, outsourcing).

The introduction of a new pathogen requires a comprehensive review of all procedures and safety measures (Clause 8.5, management of change). This includes communication to all staff regarding the new agent and the associated procedures, as well as additional training when necessary.

Clause 8.6 addresses control of critical equipment and materials, requiring scheduled maintenance activities, including HEPA filter replacement, verification of security locks, monitoring of pathogen storage freezers, and maintenance of accurate inventory records.

Clause 8.7 emphasizes training on the correct use of personal protective equipment (PPE) and requires written procedures for key processes such as pathogen storage, identification, segregation, packaging, transport, decontamination, and waste disposal.

Clause 8.9 focuses on the release of biological agents and toxins, requiring validation of inactivation and decontamination processes. Additionally, all personnel must receive training on emergency response plans.

The standard also requires implementation of cybersecurity measures, including data protection, network security, and access control, in order to prevent sabotage and ensure secure management of pathogen inventory databases.

Clause 9 concerns monitoring, measurement, analysis, and evaluation. Particular attention is given to weak signals (for example, repeated minor equipment malfunctions), which may precede major failures. Timely reporting is essential, as delays can reduce the effectiveness of corrective actions.

Clause 10 highlights the importance of continuous improvement in biosafety and biosecurity management systems. This may include the establishment of anonymous reporting systems for near-miss events, integration of laboratory emergency plans with national disaster management frameworks, and improvements in training or digital access control systems (Clause 10.1).

Clause 10.2 emphasizes the need for rapid identification of the causes of incidents or non-conformities, through audits and corrective actions, with clearly defined timelines. Clause 10.3 addresses preventive actions aimed at reducing future risks and suggests the use of PESTLE analysis (Political, Economic, Social, Technological, Legal, Environmental factors).

Finally, the standard promotes the development of a culture of responsibility, emphasizing transparency, trust, and shared accountability [90].

With regard to inventory control, the standard emphasizes accountability of pathogens and toxins, stock management, access restrictions, and traceability (audit trails). It also addresses emergency preparedness, including response to spills, containment breaches, and outbreak escalation procedures.

Particular attention is given to insider threats, through personnel reliability programs, behavioral monitoring, and awareness of dual-use issues [90]. In addition, the standard considers the broader impact of biosafety and biosecurity on public health, national security, and international obligations, such as the Biological Weapons Convention (BWC) and the International Health Regulations (IHR).

Finally, ISO 35001 promotes a strong culture of biosafety and biosecurity based on responsibility, transparency, vigilance, and trust among all stakeholders [90]. Moreover, ISO 35001 provides detailed guidance on risk assessment and highlights the importance of continuous personnel training as a key measure to prevent laboratory incidents associated with human error.

Overall, ISO 35001:2019 shares several elements with other certification systems, such as ISO 15189, ISO 17025, and ISO 9001, particularly in relation to quality management, personnel competence, and training. However, ISO 35001 places a stronger focus on biorisk management, including laboratory-acquired infections, pathogen escape, dual-use research, and insider threats.

### 3.3. Accreditation

Accreditation is the formal recognition that a laboratory or conformity assessment body is competent and impartial to perform defined activities within a specified scope [70,72]. Accreditation does not replace containment certification, and certification does not automatically demonstrate analytical competence. At the European level, Regulation (EC) No 765/2008 establishes the common framework for accreditation and requires each Member State to designate a single national accreditation body [107]. National accreditation bodies differ across jurisdictions, and the following are illustrative examples rather than an exhaustive list. Examples include Accredia in Italy, the Comité Français d’Accréditation (Cofrac) in France, and the United Kingdom Accreditation Service (UKAS) in the United Kingdom, Deutsche Akkreditierungsstelle (DAkks) in Germany, and Entidad Nacional de Acreditación (ENAC) in Spain [108,109,110]. Outside Europe, comparable bodies include the Standards Council of Canada (SCC), the National Association of Testing Authorities, Australia (NATA), South African National Accreditation System, South Africa (SANAS), the China National Accreditation Service for Conformity Assessment (CNAS) in China and ABSA in the USA [111,112,113,114]. At the international level, accreditation has historically been coordinated through the International Laboratory Accreditation Cooperation (ILAC), which links accreditation bodies operating according to ISO/IEC 17011 [72,115]. As of 1 January 2026, this international structure has been reorganized through Global Accreditation Cooperation Incorporated, established to bring together the work of ILAC and the International Accreditation Forum (IAF) [116,117].

## 4. Discussion

In this review, we have highlighted how, despite the availability of biosafety manuals and guidance for proper risk management, a non-negligible number of incidents still occur in both clinical diagnostic and research laboratories, sometimes with relevant consequences for the environment and the wider community. The available data suggest that many laboratory-acquired infections (LAIs) are still largely associated with insufficient operator training, which in turn reflects the lack of full standardization in biosafety laboratory management practices.

Certification systems may represent an effective approach to promote greater harmonization and consistency in laboratory procedures. However, not all certification schemes have the same value. Some focus on specific aspects (for example, quality assurance or technical competence), while others address similar areas but with different depths of analysis (as summarized in Table 2). Among them, ISO 35001 appears to be one of the most comprehensive frameworks, particularly with regard to staff training, which is required to be continuous and regularly updated. In addition, it establishes the obligation to inform personnel about any updates in procedures, as well as new protocols related to pathogens introduced into the laboratory. Nevertheless, in several European countries ISO 35001 is not yet widely implemented, and in some cases, there are still no accredited bodies able to perform certification audits. Furthermore, certification is generally not a prerequisite for obtaining official authorization from national authorities. Another limitation is that ISO 35001 cannot fully address differences in sample handling practices for the same pathogen across different countries. For instance, Crimean–Congo hemorrhagic fever virus (CCHFV) is classified as an RG4 pathogen by WHO and CDC, and countries such as Sweden, Switzerland, France, Germany, Italy, and the United Kingdom recommend BSL-4 conditions for diagnostic activities [118]. Conversely, other countries, including the United States, South Africa, Kazakhstan, Slovenia, and Georgia, allow diagnostic testing at BSL-3 level, whereas Bulgaria, Turkey, and Serbia recommend BSL-2 laboratories [119]. For several diagnostic approaches, with the exception of viral culture, it is also possible to inactivate samples prior to analysis, thereby reducing the biosafety level requirements. These differences highlight the absence of a universally harmonized approach even for high-risk pathogens [120,121,122].

Moreover, accreditation processes for biosafety laboratories vary considerably across countries. In some cases, licenses must be renewed annually (e.g., Canada and the Philippines), while in others renewal is required every three years, as in Australia. In contrast, some countries provide permanent licenses, as reported for England, Luxembourg, Germany, and Kyrgyzstan [123]. Only in selected cases is certification required as part of the accreditation process. For example, in Australia and France accreditation systems may be based on ISO standards such as ISO 15189, but formal certification is not mandatory [123].

Differences also exist in infrastructure requirements and oversight mechanisms. At present, there are no universally agreed criteria for construction and structural validation of high-containment laboratories [38]. For example, to our knowledge, only the BSL-4 facility in Wuhan has explicitly declared its location within an earthquake-resistant structure capable of withstanding magnitude 7 (Ritter scale) events [26] or Galveston BSL4 where the structure is built to resist a category 5 hurricane Ike [124].

Limitations: this study has several limitations. We did not provide a detailed analysis of country-specific legislation governing licensing and operation of biosafety laboratories, and therefore differences in regulatory compliance may not be fully captured. In addition, no direct consultation with national authorities was carried out, which could have provided more detailed and updated information.

Recommendation: Given that no certification system currently has the authority to verify the structural integrity of laboratory buildings or to assess in detail the specific activities conducted within individual facilities, the establishment of an independent supranational body—similar in concept to the International Atomic Energy Agency-(IAEA)—could be considered. Such an organization should include multidisciplinary expertise (biosafety, biosecurity, infrastructure engineering, national security, virology, and microbiology) and be able to evaluate infrastructure resilience, laboratory activities, and the adequacy of funding allocated for maintenance.

This approach could also contribute to addressing the lack of legislation in several countries regarding the handling of RG3 and RG4 pathogens, even in settings where BSL-3 and BSL-4 laboratories are already operational. As also highlighted in the Global BioLabs Report, not all BSL-3 facilities provide the same level of protection, and in some cases, safeguards may not be fully adequate for research involving pathogens with pandemic potential [39].

## 5. Conclusions

This review highlights that, despite the availability of international biosafety guidelines and national regulatory frameworks, substantial heterogeneity persists in the management of high-containment laboratories. Laboratory incidents continue to occur, with human error, insufficient training, and inadequate adherence to standardized procedures remaining important contributing factors. Certification systems may support greater harmonization of laboratory practices; among them, ISO 35001 provides a comprehensive framework specifically focused on biorisk management. However, its implementation remains limited, and certification is not uniformly integrated into national authorization and accreditation processes. Moreover, relevant differences persist among countries in pathogen handling, containment requirements, infrastructure standards, and laboratory oversight. Overall, greater international harmonization of biosafety and biosecurity practices is needed to ensure consistent levels of protection across BSL-3 and BSL-4 laboratories. In this perspective, the establishment of an international auditing body could represent an important step toward strengthening global health security and improving collective preparedness.

## Figures and Tables

**Table 1:** Comparative operational, containment, and structural characteristics of BSL-3 and BSL-4 laboratories.

Feature	BSL-3 (Biosafety Level 3)	BSL-4 (Biosafety Level 4)
Risk Level [16]	Moderate to high individual risk; low community risk	High individual risk; high community risk
Pathogen Types [15,16]	Serious or lethal agents	Life-threatening, exotic agents with no treatment/vaccine
Example Pathogens [15,16]	*Mycobacterium tuberculosis*, Rift Valley virus	Ebola virus, Marburg virus, Crimean-Congo hemorrhagic fever virus
Primary Barriers [16]	Biosafety Cabinets	Class III biological safety cabinets or positive-pressure suit systems
Air Handling [16,32]	Controlled directional airflow with negative pressure; HEPA filtration	Dedicated supply/exhaust air; double HEPA filtration
Facility Location [32,33]	Separate from the main corridor	Highly isolated containment zone, often located in a separate building or dedicated secure area
Personnel Access [15]	Restricted to authorized and trained personnel	Highly restricted and strictly controlled access with specific entry/exit procedures
Decontamination [15,16]	Autoclave on-site; waste treated before disposal	Full decontamination shower upon exit; chemical showers
PPE Usage (Suits & Protection) [15,16]	Required personal protective equipment includes rear-fastening laboratory coats or full-body coveralls, double gloves, dedicated footwear, and mandatory respiratory protection using either FFP3 respirators or powered air-purifying respirators (PAPRs)	Personnel must either wear a fully encapsulating, airtight positive-pressure suit (“space suit”), equipped with double- or triple-sealed gloves and integrated boots and supplied with breathable air through an external hose, or work within a Class III biosafety cabinet (glove box) while wearing standard personal protective equipment (PPE)
Entry & Exit Procedures [15,16]	Entry is through interlocking double doors (anteroom) where PPE is donned. Upon exit, PPE is removed in the anteroom, and hands are sanitized; a final shower is usually optional or recommended by protocol. Waste leaves the area after being autoclaved	Entry requires full removal of personal clothing and donning the positive-pressure suit. Exit is strictly controlled: a chemical decontamination shower is mandatory while still wearing the suit, followed by suit removal and a mandatory personal shower. All materials or waste exit only through a double-door autoclave
Seismic Risk Category [34,35]	Typically classified as Risk Category III (high hazard to public but not an absolute essential facility)	Strictly classified as Risk Category IV (Essential Facility) under the highest importance factor equations
Structural Deformation [32,33,36]	Limited plastic deformation is acceptable. Minor non-structural cracking or cosmetic damage may occur	Strictly Elastic Behavior. No permanent deformation or cracking is tolerated. Monolithic shell and liners must remain intact
Airtight Envelope Integrity [15,16]	Minor localized stress on joints is tolerated; secondary containment relies on inward airflow restoring quickly	Absolute Envelope Integrity. Internal coatings must experience zero micro-fissures
Power Continuity [35,37]	Standby backup generators activate within standard windows to restore critical ventilation	Seismic-Isolated Backup Power. Redundant generators are mounted on seismic isolators and ignite instantly
Bio-Waste Containment [15]	Holding tanks are anchored but may rely on automatic shut-off valves to prevent external flow	Isolated Effluent Decontamination System (EDS) Integrity. The EDS is independently base-isolated and fitted with chemically resistant flexible seals designed to accommodate shear displacement

**Table 2:** International Organization for Standardization (ISO) currently in use and their role in the governance of BSL-3 and BSL-4 laboratories.

Standard	Title	Role in BSL-3/BSL-4 Governance
ISO 9001:2015 [85]	Quality management systems—Requirements	Institutional quality framework; document control; process management; corrective actions; continual improvement.
ISO 14644-1:2015 [86]	Cleanrooms and associated controlled environments—Part 1: Classification of air cleanliness by particle concentration	Clean-air classification; particle monitoring; environmental qualification of controlled areas. It is relevant in BSL-3 and BSL-4 laboratories to ensure proper air filtration through HEPA filters and prevent the escape of pathogens.
ISO 17034:2016 [87]	General requirements for the competence of reference material producers	Use of traceable reference materials for assay validation, quality control, calibration, and method verification.
ISO/IEC 17011:2017 [72]	Conformity assessment—Requirements for accreditation bodies accrediting conformity assessment bodies	Oversight of bodies that accredit laboratories, inspection bodies, proficiency testing providers, and other conformity assessment organizations.
ISO/IEC 17025:2017 [88]	General requirements for the competence of testing and calibration laboratories	Accreditation of testing/calibration activities; validation of methods; equipment calibration; environmental or performance measurements supporting containment verification.
ISO 45001:2018 [89]	Occupational health and safety management systems—Requirements with guidance for use	Worker-safety governance; hazard identification; occupational risk assessment; training, awareness, and competence; emergency preparedness; health and safety performance monitoring. In biological laboratories, it is used in conjunction with ISO 35001.
ISO 35001:2019 [90]	Biorisk management for laboratories and other related organizations	Biorisk assessment; biosafety and biosecurity governance; roles and responsibilities; staff competence and training; SOPs; incident reporting; emergency preparedness; continual improvement.
ISO 14644-3:2019 [91]	Cleanrooms and associated controlled environments—Part 3: Test methods	Performance testing under as-built, at-rest, and operational conditions; airflow-related testing; environmental performance verification.
ISO 15190:2020 [92]	Medical laboratories—Requirements for safety	Safe laboratory practices; personnel protection; safety procedures; exposure prevention; laboratory safety training; integration of biosafety into medical laboratory operations.
ISO 15189:2022 [93]	Medical laboratories—Requirements for quality and competence	Diagnostic quality management in medical BSL-3/BSL-4 laboratories; sample handling; reporting reliability; competence of medical laboratory processes.
ISO/IEC 27001:2022 [94]	Information security, cybersecurity and privacy protection—Information security management systems—Requirements	Protection of pathogen inventories, sensitive research data, access-control information, biosecurity records, and dual-use or security-sensitive information.
ISO/IEC 17043:2023 [95]	Conformity assessment—General requirements for the competence of proficiency testing providers	External quality assessment; inter-laboratory comparison; verification of analytical reliability for diagnostic or research assays.
ISO/IEC 17020:2026 [96]	Conformity assessment—Requirements for bodies performing inspection	Independent inspection of facilities, installations, equipment, engineering controls, or compliance-related processes.
ISO/IEC 17024:2026 [97]	Conformity assessment—General requirements for bodies operating certification of persons	Certification schemes for individual competence, such as biosafety professionals, inspectors, auditors, or specialized technical personnel.

## Data Availability

No new data were created or analyzed in this study. Data sharing is not applicable to this article.
